# The impact of multiple interventions to reduce household exposure to second-hand tobacco smoke among women: a cluster randomized controlled trial in Kalutara district, Sri Lanka

**DOI:** 10.1186/s12889-017-4820-8

**Published:** 2017-10-16

**Authors:** A. M. A. A. P. Alagiyawanna, N. Rajapaksa –Hewageegana, N. Gunawardena

**Affiliations:** 1 Ministry of Healthcare and Indigenous Medicine, Health Promotion Bureau, No 02, Kynsey Road, Colombo, 08 Sri Lanka; 2 Department of Community Medicine, University of Colombo, No.25, PO Box, 271 Kynsey Road, Colombo-08, Colombo, Western Province Sri Lanka

**Keywords:** Second-hand smoking, Health promotion, Empowerment, Women, Randomized controlled trial, Lower-middle income country, Sri Lanka

## Abstract

**Background:**

Second-hand smoke (SHS) in households remains a serious public health problem in Sri Lanka, partly due to a lack of voluntary prohibitions of tobacco smoking inside houses. Women are especially at risk of being exposed. Effective community based interventions to reduce the SHS in households targeting women is scarce. The objective of this study was to examine the impact of a multi-component intervention on household SHS exposure among Sri Lankan women.

**Methods:**

Thirty clusters of 25 women (aged 18–65) from 750 households were randomized into the intervention and control groups. Women in the intervention group were exposed to activities which focused on improving knowledge on the health effects of SHS, attitudes towards SHS exposure, right to a smoke-free living and women empowerment against smoking. The duration of the intervention was six months. The comparison group received no intervention. The primary outcome of interest was self-reported SHS exposure in the household within 7 days prior to data collection. The secondary outcomes were exposure in the past 30 days, knowledge of the health risks of exposure, attitudes towards exposure, right to smoke-free living, women empowerment against smoking, and smoking inside the homes.

**Results:**

Final assessment was in 329 (89.6%) in the intervention group and 309 (85.8%) in the comparison group. Following the intervention, significantly lower proportion of women in the intervention group as compared to the control group reported SHS exposure in their households within 7-days (9.2% vs. 15.3%, *p* = 0.02) and 30-days (13.6% vs. 21.6%, *p* = 0.008) prior to the post survey. As compared to the control group, significantly higher median scores were observed in the intervention group on the knowledge of the health risks of exposure to SHS (*p* < 0.001), attitudes on exposure to SHS (*p* = 0.004), right to smoke free living (*p* = 0.001) and women empowerment (*p* < 0.001).

**Conclusion:**

Multi-component intervention activities were effective in reducing household exposure to SHS among women.

**Trial registration:**

Sri Lanka Clinical Trials Registry SLCTR/2014/033.

**Electronic supplementary material:**

The online version of this article (10.1186/s12889-017-4820-8) contains supplementary material, which is available to authorized users.

## Background

Environmental tobacco smoke or SHS is a serious health hazard and the smoke contains more than 7000 chemicals. At least 250 of such chemicals are known to be harmful, with hydrogen cyanide, carbon monoxide, and ammonia being a few examples [1]. It is associated with increased risk of cardiovascular disease, chronic respiratory illness, including lung cancer and nasal cancer among adults [2].

The Tobacco and Alcohol Act, No. 27 of 2006, practiced presently in Sri Lanka, has provisions for smoke free environments in health care facilities, all educational facilities, all governmental facilities, as well as in indoor private offices and workplaces [3]. Furthermore, Sri Lanka became a signatory to the World Health Organization (WHO) Framework Convention on Tobacco Control in September 2003 and ratified the same in November 2003 [4]. It, thus, obligates the country to implement its article 8, ‘protection from exposure to SHS’ [5]. However, anti-smoking law in Sri Lanka does not cover smoking inside homes. Thus, additional measures are required to protect non-smokers, particularly women and children, from SHS exposure. Women not having the power to ban smoking in their own homes is also a discrimination against them. Despite an obligation to conventions to protect women against SHS, specific legislation to control tobacco use inside homes is not likely to be imposed in Sri Lanka because of complex social and cultural issues. De-normalizing tobacco use and normalizing smoke-free homes are therefore more likely solution, which has to be realized from the grassroots level. Such measures would also ensure non-smoking women’s rights to the enjoyment of the highest attainable standard of physical and mental health [6].

Sri Lanka records very low rates of current tobacco smoking among women (0.1%) compared to males (29.4%) [7]. Women inhale SHS in their homes due to tobacco use of males. Exposure to SHS remains a significant problem [8] in Sri Lanka despite the recent laws prohibiting tobacco smoking in public places. Though the previous studies show a decline of the prevalence of exposure to SHS (within 7 days) at home from 21.1% in 2006 to 10.8% in 2011 [9], the latest non-communicable diseases, risk factor survey in 2015 indicates that 21.6% of females of 18–69 years were exposed to SHS at home during a 30 day period [7].

The community based initiatives and interventions to develop smoke free homes have been conducted in many countries, with mixed success [10–16]. It has been shown that the intervention programmes when implemented along with population-based strategies (such as public education campaigns on SHS in homes, laws enforcing 100% smoke-free public places and workplace initiatives for smoke free homes) are more effective than individually carried out programs [17]. Numerous calls for action on women and tobacco have highlighted the need for intervention on SHS exposure among women [16, 18, 19] but the studies on community based interventions are limited from lower middle and lower income countries [12–14]. Most of the existing interventions were targeted to pregnant women or women with children [10, 12, 20]. A quasi experimental study from India to develop smoke free homes was found to be effective following a six month multiple intervention period targeting women and community leaders [13]. Another quasi experimental study to increase the number of smoke free homes in Pakistan had attempted multiple activities in school and community [14]. The results revealed that smoke-free homes increased from 43% (95%CI 37.4–48.2) to 85% (95%CI 80.9–89.2) following three months of intervention. A similar attempt in the United Kingdom also found a significant (*p* < 0.0001) increase in smoke free homes from 35% at baseline to 68% six months of the post-implementation of intervention [11]. Multiple strategies and activities integrated with each other and at different levels (individual level, family level, community level, institutional level and policy level) are more effective in health promotion interventions [21]. We could not find any community based Randomized Controlled Trial (RCT) on SHS reduction at home among women in lower and lower middle income countries. The interventional studies to reduce exposure to SHS at home have not been reported from Sri Lanka.

Exposure to SHS can be measured objectively using biomarkers such as saliva or urinary cotinine or environmental indoor measurement of tobacco smoke constituents such as air nicotine or particulate matter [22]. Questionnaire was the most commonly used method to assess SHS exposure [23] and the most commonly used indicator to ascertain the exposure was the presence of smokers [24]. Moreover, a strong correlation was found between self reported SHS exposure and urinary cotinine level [25]. In this backdrop, the objective of the present study was to implement and evaluate an intervention comprising multiple interventional activities to reduce household exposure to SHS among women. The hypothesis of this study was whether multiple interventions would be effective to reduce household exposure to SHS among women.

## Methods

The present study is a cluster randomised controlled trial, implemented and reported in accordance with the Consolidated Standards of Reporting Trials (CONSORT) statement [26] and its extension to cluster randomised trials [27]. It was conducted between June to December 2015. This is a part of a wider programme on promoting the right to smoke-free living for Sri Lankan women. The total duration of the intervention was six months (until the post intervention survey).

### Study setting and participants

This study was conducted in purposefully selected three Medical Officer of Health (MOH) areas (Agalawatta, Bulathsinghala, Ingiriya) in Kalutara district, which is one of the 25 districts in Sri Lanka. Kalutara district was selected due to its close proximity to Colombo (where the principle investigator was based). The selected MOH areas were adjacent to each other. A Grama Niladhari (GN) division, which is the smallest administrative unit in Sri Lanka, was considered as a cluster. The study population consists of women aged 18–65 years and a group of 25 women were recruited for a cluster. If a woman was found to be a smoker, she was excluded from the study.

### Sample size

The sample size estimation was done comparing the proportion of women exposed to SHS between the intervention and control groups. This is related to the objective of the study. The number of clusters required per group was assumed as *c* and the cluster size was decided as 25 women. In the estimation of sample size, we calculated the number of clusters required per group, according to the formula for cluster randomized trial [28]. If *n* individuals are sampled in each cluster, and *c*, the number of clusters required, is given by,

$$ c=1+{\left({z}_{\alpha /2}+{z}_{\beta}\right)}^2\left[{\pi}_0\left(1-{\pi}_0\right)/n+{\pi}_1\left(1-{\pi}_1\right)/n+{k}^2\left({\pi_0}^2+{\pi_1}^2\right)\right]/{\left({\pi}_0-{\pi}_1\right)}^2 $$

Where *π*_1_and *π*_0_are the true proportions in the presence and absence of the intervention, with *k* being the coefficient of variation of true proportions between clusters within each group.

The proportion of those who were exposed to SHS in the control clusters was estimated as 21.6%, considering the latest 2015 Sri Lankan data (7) on SHS (*π*_0_= 0.11). In the absence of empirical data to estimate *k*, 0.3 was adopted as *k* to imply that the true rates in the control clusters would vary roughly between *π*_0_(1± 2 *k*). In this study, we assumed that the intervention would reduce the proportion of women exposed to SHS by 10%, estimating the *π*_1_ as 11.6% (0.12). The estimated value of *z*_*α*/2_ was 1.96 corresponding to the level of significance of *p* = 0.05 and estimated value for *z*_*β*_was 0.84 corresponding to the power of 80%. With these data, *c* was estimated to be 15.

### Selection of GN areas and randomization

In the first stage of sampling, a list was made of all GN divisions in the three MOH areas alphabetically and assigned consecutive numbers. From the list, we selected 30 GN divisions randomly. If the GN division selected was located within 5 km of any GN division, that was already selected, we replaced that GN division by another. Of the selected GN divisions, half was randomly assigned to the intervention group and the other half to the control group.

### Selection of households in a cluster

The selection of women in a cluster was done by household visits in the field at the time of data collection. Within the GN division, starting point of the survey was a random point selected by dropping a pin onto a map of the area. Commencing from that point, households were enrolled according to a pre-determined direction (right) until 25 houses with eligible women was identified. A similar procedure was adopted to select the women in the control group from the three MOH areas. If more than one eligible woman was present in the selected household, only one was selected randomly to be the study unit.

### Primary outcome measure and the operational definition

The primary outcome of the study was women with recent exposure to SHS in their households. Recent exposure to SHS was defined in this study as the exposure to tobacco (cigarette, bidi/cigars) smoke inside their household within last 7 days. This was ascertained by self-reports of the participants.

### Secondary outcomes

The secondary outcomes were:

- exposure of women to SHS within the last 30 days prior to the data collection
- knowledge of women on health risk of exposure to SHS
- attitudes of women towards exposure to SHS, right to smoke free living and women empowerment against smoking
- Observed evidence on smoking inside houses

### Outcome measurements

Data were collected using a paper-based, close ended response interviewer administered questionnaire and an observational checklist by the data collectors. The questionnaire assessed the status of exposure of women to SHS in their households within 7 and 30 days prior to data collection. It also assessed the other secondary outcomes. It included relevant questions from previously valid and reliable questionnaires from other studies [22, 24, 29–36] and questions from expert opinion. The initial draft of the questionnaire had 67 items and it was subjected to content validity by a panel of five experts involving two Consultant, Community Physicians who were experts in the research methodology and management of community programs, a Pulmonologist, a Statistician and a Sociologist. The final study instrument consisted of 40 items. It was divided into seven sections. One section consisted of seven items on socio-demographic information about the participants, the second had two questions for exposure to SHS, third consisted 13 questions for knowledge on the health risk of exposure to SHS. Five statements on the attitude of respondents towards exposure to SHS was in the fourth section whilst three statements about right to smoke free living were in the fifth section. Last two parts had four statements for women empowerment against smoking. A checklist containing six areas of observation was developed to observe the evidence on smoking inside houses. The English version of the questionnaire was then translated into Sinhala and Tamil, and then back to English in accordance to the standard procedures for questionnaire translation [37].

To ensure that the questionnaire was suitable for women in the community, it was pretested with 20 women in a GN area, which was not included in the study. The amendments proposed by the participants in the questionnaire were incorporated. The reliability coefficient of the questionnaire was calculated by using SPSS v.20. The Cronbach’s alpha value of 0.74, 0.70, 0.78 and 0.71 was computed from knowledge of health risks of exposure to SHS, attitude on exposure to SHS, right to smoke free living and women empowerment sections respectively. The checklist was also pretested in the 20 households, and was found to be suitable.

### Development of intervention

The principle investigator reviewed literature to identify the effective interventions to reduce household exposure to SHS among women. In addition, eight in-depth interviews were held with five public health experts, two psychiatrists, and a sociologist. The health belief model [38], social cognitive theory [39] and existing evidence-based SHS prevention interventions [40–53] guided the intervention design. Intervention strategies included persuasion, skill development, role modelling, empowerment, cues to action, environmental cues and reinforcement of actions taken to create smoke free homes. Multi- component intervention activities were targeted to women in households in the intervention GN divisions focussing on health effects of exposure to SHS, attitudes of women towards exposure to SHS, right to smoke free living and women empowerment against smoking.

**Table:** 

**Household exposure to SHS among women- Theoretical framework** **Reinforcement:** reinforcing the knowledge of the women on the health effects of SHS and third hand smoking, positive attitudes towards right to smoke free living and environmental factors (social norms, role models, social support, opportunities). **Perceived susceptibility:** belief of the women that even though they have no symptoms due to SHS, they are susceptible to short and long term health effects of SHS. **Cues to action:** display of posters and stickers might encourage women to maintain smoke free homes. **Expectation, benefits and barriers:** learn how smoke free homes benefit them and their families and to overcome barriers of making a household SHS free. **Empowerment:** empowering women to exercise their right to smoke free living. Develop social support group against SHS within their locality. Schoolchildren to act as change agents to educate their mothers/women in their households and declare smoke free homes. **Observational learning:** select role models of women living in smoke free houses within their GN area. **Behavioral capacity:** improve skills of women on the actions to be taken if somebody smokes inside the home and to exercise their right to smoke free living. **Self-efficacy:** volunteers and Public Health Midwives (PHMs) encourage women to set targets to achieve smoke free homes and in turn to accept right to smoke free living as a value and social norm.	

**Interventions and implementation** ***Training of field staff who provide services to residents of the GN divisions*** Three training workshops were organized at Bulathsinghala MOH office for the field workers of health, non-health and volunteers of all the GN divisions who were selected for the intervention. The importance of declaring the houses of the study population as smoke free homes were targeted. The field staff who provide services to adults by means of home visits or in clinics/offices were chosen based on their opportunities to interact with the women to provide the knowledge. The health staff included MOH, PHM, Public Health Inspectors, and non-health staff included Grama Niladari, Samurdi Officer, Social Service Officer and health volunteers. Additionally, the training was aimed at gaining support of the officers for a larger research. We used adult training methods such as lecture discussion, group work, group discussion, role play, experience sharing sessions in the workshop. A video developed by the Ministry of Health on health effects of SHS was also shown to them. The Principle Investigator conducted the training workshops along with a legal officer for a non-governmental organization called Alcohol and Drug Information Centre who deals against tobacco and alcohol. ***Individual and group health education sessions for women.*** Intervention was in the form of individual and interactive small group health education sessions. Two trained volunteers were selected from each cluster in a GN division and were trained to deliver the health education sessions. Selected volunteers were the active members of the mother support group, which is a strong volunteer organization to strengthen the community level health promotion activities in Sri Lanka. Each volunteer was asked to visit the half of the households [12, 13] of the group of women selected from a GN division for the intervention. Intervention was done with the PHM who was also trained to deliver the intervention as described earlier. The volunteers organized small group discussions with the women and invited the area PHM. The area PHM initiated discussion on family wellbeing as an entry point in the discussion and thereafter conducted the discussion to identify the problems faced by the women in relation to being exposed to SHS. Women were educated how to apply avoidance behaviour when exposure to SHS, such as walking away from SHS, showing displeasure against exposure to SHS. Identified problems were prioritized and the two-trained volunteers with inputs from the women and the area PHM developed a problem and solution tree. Special attention was given to educate women on health effects of exposure to SHS and the right to smoke free living. Leaflets and stickers were distributed among women. They were persuaded to implement 100% smoke free environment in their homes. The women were guided to decide how they could reduce exposure to SHS, as applicable to their own home. Modalities like demonstration, role-playing, storytelling and sharing experience were used to educate and motivate them to initiate activities. Each group discussion lasted for about one and a half hours. The two health volunteers noted down the interventional activities selected by women. Those included discussions with their spouses on health effects of passive smoking, SHS exposure avoidance behaviour, and display stickers on “this house is tobacco smoke free”. In addition, women themselves initiated some activities, which were not directly related to SHS reduction, but improved family well-being by strengthening family bond. They were household money management, home gardening, proper garbage disposal, hygienically safe kitchen, family dinners and religious activities, etc. When women became empowered by creating changes within their own home, they visited neighborhood homes too, to explore the possibility of spreading the results to the community away from the household. Initially PHM and the volunteers motivated them, later they themselves disseminated the results to the community. Some community level activities initiated by the women were evident, though not formally assessed. In most instances, other family members (children, husband and parents) and volunteers also made an effort to get the neighboring households involved. Initially, the volunteers visited the allocated houses once in a fortnight and gradually reduced the visits when the activities were established. The volunteers’ arranged group meetings with the women in the selected households once a month in the first three months, followed by once in every one and a half months. ***Distribution of educational materials*** Education materials (posters, leaflets and short video clips) were also distributed to community clinic centers, local shops, religious centers, preschools, and schools in the selected GN divisions. ***School based intervention to train the*** **schoolchildren to be change agents** The objective of the school-based intervention was to improve students’ knowledge of health effects of exposure to SHS and the right to smoke free living and to use them as change agents to educate their mothers/women in their households and declare smoke free homes. The schoolchildren in grades 8–10 of six secondary schools in the intervention GN divisions were selected for this purpose. The Public Health Inspectors who received the training as a part of the same intervention delivered two 45 min sessions over two days. The duration of these sessions is consistent with regular school lessons. Teaching methods included active learning activities such as lecture discussions, role-playing and storytelling. All programs were delivered to students in their usual classroom setting, during school hours. In addition, all the students in six schools were invited to participate in a poster competition to promote the message “right to smoke free living”.	

### Data collection

For the assessment of outcomes and other related factors in both intervention and control group of women of the selected GN divisions, baseline survey was conducted prior to the intervention, and follow-up survey was conducted immediately after the completion of the intervention. The questionnaire and the checklist were completed by the data collectors who were not involved in the intervention by doing the household visits, before the intervention and after the intervention. To ensure that women could honestly provide their responses, women were pre-informed about the confidential nature of their responses. The questionnaire was given first and then the observational checklist was completed.

### Fidelity

The health volunteers in terms of how much of the intended intervention was actually delivered, assessed the fidelity of the intervention. The number of participants among the intervention group for each small group discussion and number of visits done by them to intervention houses during the period of intervention were the indicators used.

### Statistical analysis

The collected data were entered into epi-data 3.1 software [54] and analysed using SPSS 20.0 [55]. Between group and within group comparisons were done using parametric and non-parametric tests (Paired t test, Independent t test, Chi-Square test, McNemar test, Wilcoxon Signed Ranks test and Mann-Whitney U test). Comparison of the basic characteristics of the completers and non-completers were also tested.

The primary outcome indicator, the proportion of women with recent exposure to SHS at home was estimated by taking the number of women who report anyone smoking tobacco (cigarette bidi / cigars) inside the household within last 7 days in the presence of these women as the numerator and all the women in the study group as the denominator. This proportion was estimated for both intervention and control groups prior to and after the interventions. Secondary outcome of exposure to SHS was analyzed using the same numerator and denominator, but with the number of women exposed within last 30 days.

The secondary outcome of knowledge of women on the health risk of exposure to SHS was assessed using questions with *yes*, *no* or *do not know* responses. Using a scoring system, each woman was awarded one point for each correct answer while incorrect, blank and do not know the answers were awarded zero points. The total score for this scale ranged from 0 to 13, with a higher score representing a greater knowledge. Attitude of women towards exposure to SHS, right to smoke free living and women empowerment against smoking were also assessed using relevant statements in a five point Likert scale format, with responses ranging from *strongly disagree* to *strongly agree* with middle neutral. The scoring system used was assigned a score of 0–4 for each statement in which a score of zero was assigned to *strongly disagree* and a score of four for *strongly agree* with other responses for positively worded items, and a reverse scoring system for negatively worded items. Scores were summed to obtain a total score for attitude of women towards exposure to SHS, attitude on the right to smoke free living and attitude on women empowerment that ranged from 0 to 20, 0–12 and 0–16 respectively. The median scores for knowledge of women on the health risk of exposure to SHS, attitude of woman’s exposure to SHS, right to smoke free living and women empowerment against smoking were calculated for both intervention and control groups prior to and after the interventions. In analysis, the scores for the knowledge, attitude towards exposure to SHS, right to smoke free living, women empowerment were tested for normality of distribution using one sample Kolmogorov-Smirnov test.

The secondary outcome of observed evidence on smoking inside houses was assessed using the checklist mentioned above. Presence of at least two out of the six was considered as evidence of smoking inside the house. The proportion of houses with or without evidence of smoking was compared using Chi Square test and Mc Nemar tests. Cluster analysis was also conducted between intervention and control group before and after the intervention. The differences in the pre and post results of the primary and secondary outcomes of the intervention and control groups were compared and assessed for statistical significance. A value of 0.05 or less was considered as statistically significant. A significant improvement of the outcome in the intervention in comparison to the control group was taken as an evidence of effectiveness of the multi-component intervention activities to reduce household exposure to SHS among women.

## Results

**Fig. 1:**
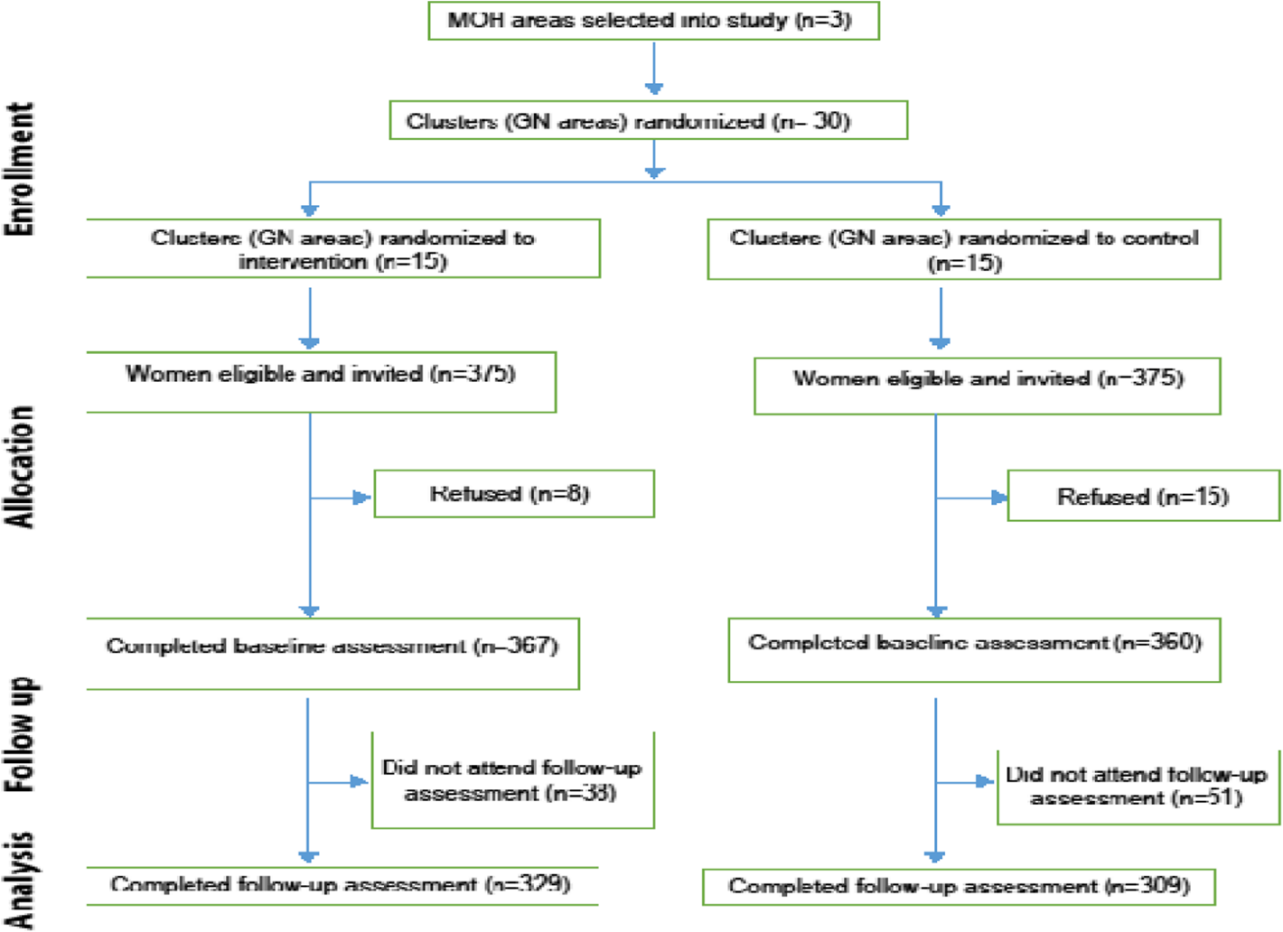
Study flow diagram

The Fig. 1 shows the study flow diagram showing the number of clusters and participants at each phase of the trial, according to the CONSORT statement 2010 for cluster randomized trial [27]

### Participation and completion rate

Thirty GN areas were randomized to either the intervention (15 GN divisions) or control (15 GN divisions), and 750 eligible households (375 intervention and 375 control) were selected and invited to the baseline survey. Twenty-three were removed due to refusal to participate (8 intervention and 15 control). Of the 727 women who completed the baseline survey (367 intervention and 360 control), 89 (12.2% non-response) did not attend the follow-up survey at six months, leaving 638 women (329 intervention and 309 control) for completer analysis. Households of all who completed the baseline survey were visited at least twice before being classed as ‘non-completer’ and the reasons recorded for this included moving house or moving out of the house for employment.

### Fidelity

Health volunteers recorded the number of participants for each small group discussion and number of visits done by them to intervention houses during the period of intervention. Between 80 and 90% attended in each small group discussion and more than 90% of planned home visits were done.

### Completers and non-completers

Compared with the participants who attended the follow-up survey (completers), those who did not attend it (non-completers) were more likely to be employed (31.7% vs. 53.9%, *p* < 0.001). The two groups were similar in terms of other socio-demographic factors, knowledge of health risks of exposure to SHS, attitude related variables and observed evidence on smoking inside the home (Additional file 1: Table S1).

### Baseline characteristics

The intervention and control groups were similar in basic socio-demographic characteristics in terms of their ability to influence the household exposure to SHS, other than the intervention (Table 1). Mean (SD) age was 40.4 years (10.5) in the intervention group and 39.9 years (9.2) in the control group (*p* = 0.55). In both groups, more than 95% were residing in rural areas and the rest were in estates (*p* = 0.57), nearly 88% were Sinhalese (*p* = 0.75), more than half completed junior high school (*p* = 0.43), nearly 70% were housewives (*p* = 0.32), and nearly 45% had a household income less than Rs.20, 000 per month (*p* = 0.73).

**Table 1:** Comparison of the socio-demographic characteristics of study participants

		Intervention group (*n* = 329)	Control group (*n* = 309)	Significance
Age in years mean (SD)		40.4 (± 10.5)	39.9 (± 9.2)	0.55
Place of residence n(%)	Rural	316 (96)	294 (95.1)	χ^2^ = 0.31,df = 1 *p* = 0.57
	Estate	13 (4.0)	15 (4.9)	
Ethnicity n(%)	Sinhalese	291 (88.4)	274 (88.7)	χ^2^ = 0.56,df = 2 *p* = 0.75
	Tamil	8 (2.4)	10 (3.2)	
	Muslim	30 (9.1)	25 (8.1)	
Education n(%)	No schooling	19 (5.8)	16 (5.2)	χ^2^ = 2.75,df = 3 *p* = 0.43
	Primary level (Grade 1–5)	47 (14.3)	58 (18.8)	
	Junior high school	179 (54.4)	166 (53.7)	
	High school or higher	84 (25.5)	69 (22.3)	
Occupation n(%)	Housewife	219 (66.6)	217 (70.2)	χ^2^ = 0.98,df = 1 *p* = 0.32
	Employed	110 (33.4)	92 (29.8)	
Monthly income SLR n(%)	up to 20,000	153 (46.5)	134 (43.4)	χ^2^ = 0.63,df = 2 *p* = 0.73
	20,001–40,000	161 (48.9)	160 (51.8)	
	>40,000	15 (4.6)	12 (4.9)	

*χ* ^*2*^Chi-square value, *df* degree of freedom, *p* significance between intervention group and control group

### Exposure to SHS

Table 2 shows the proportion of women exposed to SHS in the intervention and control group before and after the intervention. The proportion of women exposed to SHS at home within the last 7 days before the data collection was similar (*p* = 0.58) in both intervention (19.0%) and control groups (17.3%). Following the intervention, a significantly lower (*p* = 0.02) proportion of women were exposed to SHS in their households within the 7 days prior to the post survey in the intervention group (9.2%) than the control group (15.3%). The proportions of women exposed to SHS at home within the last 30 days before the data collection was similar to 24.3% in the intervention group and 22.9% in the control group (*p* = 0.67). After the intervention, significantly lower proportion of women exposed to SHS in the intervention group (13.6%) than the control group (21.6%) in the corresponding period (*p* = 0.008).

**Table 2:** Comparison of proportions exposed to SHS at their home

Outcomes	Intervention group (*n* = 329)		Control group (*n* = 309)		Significance
	Pre	Post	Pre	Post	
	*n* (%) significance		*n* (%) significance		
Exposure to SHS in their households within last 7 days	62 (19.0)	30 (9.2)	53 (17.3)	47 (15.3)	pre p_2_ = 0.58
					post p_2_ = 0.02
	p_1_ < 0.001		p_1_ = 0.51		
Exposure to SHS in their households within last 30 days	79 (24.3)	44 (13.6)	70 (22.9)	65 (21.6)	pre p_2_ = 0.67
					post p_2_ = 0.008
	p_1_ < 0.001		p_1_ = 0.70		

Data missing: intervention (pre3, post 3), control (pre 3,post 2)

p_1_ = McNemar test, between pre and post intervention group and control group

p_2_ = Chi Square tests, between intervention group and control group pre and post

### Knowledge and attitudes of women related to SHS exposure in the household

The median difference between the two groups on knowledge of health risks of exposure to SHS, attitude towards exposure to SHS, right to smoke free living, women empowerment at households was tested for significance and the results are shown in Table 3. The scores for the above items were tested for normality of distribution using one sample Kolmogorov-Smirnov test and were <0.05 in each. Therefore, Wilcoxon Signed Ranks test and Mann-Whitney U test was used to calculate within group and between group differences respectively. None of the above variables were found to be significantly different between intervention and the control group before the intervention. A significantly higher median scores were observed on knowledge of health risks of exposure to SHS (*p* < 0.001), attitude on exposure to SHS (*p* = 0.004), right to smoke free living (*p* = 0.001), women empowerment (p < 0.001) in the intervention group compared to the control group after the intervention.

**Table 3:** Comparison of median values of knowledge and attitudes related to SHS

Outcomes	Intervention group (*n* = 329)		Control group (*n* = 309)		Significance
	Pre	Post	Pre	Post	
	Median significance		Median significance		
Knowledge on health risk of exposure to SHS	9	11.0	9.0	10.0	Pre p_2_ = 0.67
					post p_2_ < 0.001
	p_1_ < 0.001		p_1_ < 0.001		
Attitude on exposure to SHS	11	12.0	11.0	11.0	pre p_2_ = 0.12
					Post p_2_ = 0.004
	p_1_ < 0.001		p_1_ < 0.001		
Attitudes on right to smoke free living	5.0	5.0	5.0	5.0	pre p_2_ = 0.32
					post p_2_ = 0.001
	p_1_ < 0.001		p_1_ < 0.001		
Attitudes on women empowerment against SHS	5	8.0	5.0	6.0	pre p_2_ = 0.98
					post p_2_ < 0.001
	p_1_ < 0.001		p_1_ < 0.001		

p_1_ = Wilcoxon Signed Ranks Test, between pre and post intervention group and control group

p_2_ = Mann-Whitney U Test, between intervention group and control group pre and post

### Observed evidence of smoking

Observed evidence of smoking among women exposed and none exposed to SHS within 7 days of data collection is depicted in Table 4. The proportion of observed evidence of smoking in houses were apparently similar in both intervention and control groups (87.1% and 92.5%) in the exposed group before the intervention. In the exposed group, the proportion of houses with observed evidence of smoking was significantly reduced in the intervention group (63.3%) compared to the control group (85.1%) after the intervention (*p* = 0.03). After the intervention, the proportion of houses with observed evidence of smoking in the SHS exposed group was significantly reduced in the intervention group (*p* = 0.008).

**Table 4:** Observed evidence of smoking

Exposed to SHS within 7 days	Intervention group (*n* = 329)		Control group (*n* = 309)		Significance
	Pre *n* (%)	Post *n *(%)	Pre *n* (%)	Post *n* (%)	
Exposed					
Observed evidence present	54 (87.1)	19 (63.3)	49 (92.5)	40 (85.1)	Pre p_2_ = 0.35 Post p_2_ = 0.03
Observed evidence not present	8 (12.9)	11 (36.7)	4 (7.5)	7 (14.9)	
	p_1_ = 0.008		p_1_ = 0.24		
Not exposed					
Observed evidence present	12 (4.5)	6 (2.0)	9 (3.6)	11 (4.2)	Pre p_2_ = 0.57 Post p_2_ = 0.13
Observed evidence not present	252 (95.8)	290 (98.0)	244 (96.4)	249 (95.8)	
	p_1_ = 0.09		p_1_ = 0.69		

Data missing intervention (pre3, post 3), control (pre 3, post 2)

p_1_ = McNemar test, between pre and post intervention group and control group

p_2_ = Chi Square tests, between intervention group and control group pre and post

The cluster difference was also calculated and depicted in Additional file 2: Table S2. The cluster difference was not observed between the two groups at baseline in terms of exposure to SHS in their household within 7 days and within 30 days, knowledge of health risks of exposure to SHS, attitude towards exposure to SHS, the right to smoke free living and women empowerment. In contrast, a significant cluster difference was observed between the two groups at follow-up with regard to the exposure to SHS in their household within 7 days (*p* = 0.05) and within 30 days (*p* = 0.04), knowledge of health risks of exposure to SHS (*p* = 0.004), attitude towards exposure to SHS (*p* = 0.04), right to smoke free living (*p* = 0.02) and women empowerment (*p* = 0.008).

## Discussion

In this community based cluster randomized trial among Sri Lankan women, the intervention group showed a statistically significant reduction of exposure to SHS compared with the control group who did not receive any intervention. Furthermore, knowledge of the health risks of exposure to SHS, attitude of women towards exposure to SHS, right to smoke free living and women empowerment against smoking had significantly improved among women in the intervention group compared to the control group after the intervention. A significant reduction of the proportion of houses with observed evidence of smoking was evident among those exposed to SHS in the intervention group compared to the control group after the intervention. A significant difference was also observed at follow-up between intervention and control clusters in terms of primary and secondary outcomes. The present study is unique in that multi-component intervention activities were targeted through existing resources. All these can be taken as evidence of success to reduce household exposure to second-hand tobacco smoke among women directly and indirectly at individual, family, community and institutional level. The use of existing health resources will ensure sustainability as well as the acceptability of the intervention. To our knowledge, this is the first community based behavioral study among women on household exposure to SHS to use a cluster-randomized design conducted and analysed in accordance with CONSORT guidelines [26, 27]. This study design eliminated the problem of contamination, which is important for an intervention that has a strong social component. The study design incorporated a highly conservative data analysis strategy to account for clustering effect by comparing cluster summaries in addition to analysing individual values. This study had a high participation rate and low attrition.

The present study applied health belief model, social cognitive theory and existing community based SHS interventions to design the intervention. Intervention strategies identified by previous smoke free home interventions were also guided by the health belief model and the social cognitive theory [56, 57]. The theories helped to understand the causal paths that would affect the intervention. Therefore, the intervention model focused on perceptions of women, empowering them by disseminating to them the knowledge of passive smoking and cancer and other illnesses, and the skills to interact with smokers in their presence.

The present study did not find a significant difference between completers and non-completers except the occupational status of the participants. A significant proportion of women among non-completers were employed compared to completers (*p* < 0.001). If one is considering that being employed is likely to be associated with inputs related to adverse effects of exposures to SHS, having a higher proportion of non-completers in the intervention group strengthen the study results attributing it to the interventional activities.

Our results strengthen and extend findings from previous interventions on the initiatives of smoke free homes. Previous interventions done in neighboring countries (India and Pakistan) concluded significant improvement in smoke free home development three to six months of multiple interventions [10, 13, 14]. A study done in UK also concluded positive results following six month intervention [11]. The present study adds to the evidence of multiple activities at different levels to reduce the risk of exposure to SHS in a lower-middle income country, where the resources are scarce for community-based interventions.

We consider that the results of the study can be generalized to the communities with similar socio-demographic characteristics for the present study. However, several limitations of the present study should be acknowledged. The critical limit of the study is the lack of an objective measure (e.g., salivary cotinine levels) to validate self-reported exposure to SHS. Such laboratory-based measurements are not available for research purposes in Sri Lanka. Recall bias in the 30-day measure could have caused the controls less likely to recall the exposure than the cases. This could have affected the study by the overestimation of the differences of the post intervention SHS exposure between the groups. Having realized the possibility of recall bias with the 30-day measure, we used also the 7-day measure. In addition, we included secondary outcomes (knowledge, attitude, right to smoke free living, women empowerment) to supplement the evidence of the primary outcome. Observational checklist was also included to overcome the limitation of self-reports and recall bias. However, self-reports have been demonstrated to be accurate provided confidentially is assured [58]. In addition, previous studies have shown a strong correlation between levels of self-reported smoking restrictions and exposure to SHS measured by cotinine levels [25].

In keeping with the principles of research, the same questionnaire was administered before and after the intervention by interviewers. However, this could potentially introduce a bias (i.e., social desirability bias) in terms of responses about the subjective SHS exposure or knowledge and attitudes related to SHS in the houses in the post-intervention interview. The responders of the intervention group may have inclined to give what they perceive as socially acceptable answers, as they have been recently (within 6 months) involved in the multiple activities of the project [59, 60]. This could have caused an overestimate of the differences in the outcomes shown in the study. There is consistent population-level evidence that a smoke-free home is associated with increased smoking cessation and decreased cigarette consumption in adult smokers [61–66]. In the present study, the prevalence of smoking and smoking consumption in the households of the study subjects was not assessed. The present study has six-month intervention and observation period, and the long-term effect of this programme is unclear.

This research ascertained the impact of described multi-component intervention activities as a whole and did not assess the relative importance among the different intervention activities. Considering the importance of this information, it is suggested that future research on this subject should be designed to identify one single intervention that can make an impact.

## Conclusion

This six-month cluster randomized trial on the impact of multi-component intervention activities to reduce household exposure to second-hand tobacco smoke among women in Sri Lanka showed that they were effective in reducing exposure of the women to SHS at households. The study has established the feasibility of multi-component intervention activities in a lower-middle income South Asian setting. Scaling up the interventions will require assessment of cost effectiveness and long-term sustainability. Further research should investigate the influence of reduction of SHS at household on smoking cessation among adults.

## Additional files

Additional file 1: Table S1. Baseline characteristics of completers and non-completers. (DOCX 23 kb)

Additional file 2:Table S2. Analysis of difference for primary and secondary outcomes between intervention and control clusters. (DOCX 14 kb)

## Abbreviations

CI: confidence interval
GN: Grama Niladari
MOH: Medical Officer of Health
PHM: Public Health Midwife
RCT: Randomized controlled trial
SD: standard deviation
SHS: second hand smoking
WHO: World Health Organization
