# Supplementary material for: The impact of multiple interventions to reduce household exposure to second-hand tobacco smoke among women: a cluster randomized controlled trial in Kalutara district, Sri Lanka
Source: BMC Public Health. 2017 Oct 16;17:810. doi: 10.1186/s12889-017-4820-8 (PMC5644088; doi:10.1186/s12889-017-4820-8)
Supplement: Supplementary file 1 — Baseline characteristics of completers and non-completers. (DOCX 23 kb) [file 12889_2017_4820_MOESM1_ESM.docx]

**Additional file 1**

**Table S1. Baseline characteristics of completers and non-completers**

| **Variable** | **Completers (n=638)** | **Non-completers (n= 86)** | **significance** |
| --- | --- | --- | --- |
| Age in years mean (SD) | 40.17 (9.9) | 42.25(11.2) | 0.06 |
| Place of residence n (%) |  |  |  |
| Rural  Estate | 610 (95.6) | 87(97.8) | χ²=0.90, df=1  p₁= 0.34 |
|  | 28 (4.4) | 2(2.2) |  |
| Ethnicity n (%) |  |  |  |
| Sinhalese  Tamil  Muslim | 565 (88.6) | 80(89.9) | χ²=0.16, df=2  p₁= 0.92 |
|  | 18(2.8) | 2(2.2) |  |
|  | 55(8.6) | 7(7.9) |  |
| Education n (%) |  |  |  |
| No schooling  Primary level (Grade 1-5)  Junior high school  High school or higher | 35(5.5) | 4(4.5) | χ²=4.15  df=3  p₁= 0.24 |
|  | 105(16.5) | 11(12.40 |  |
|  | 345(54.1) | 44(49.4) |  |
|  | 153(24.0) | 30(33.7) |  |
| Occupation n (%) |  |  |  |
| Employed  Housewife | 202(31.7) | 48(53.9) | χ²=17.17, df=1  p₁< 0.001 |
|  | 436(68.3) | 41(46.1) |  |
| Monthly income SLR n (%) |  |  |  |
| up to 20,000  20,001-40,000  >40,000 | 287(45.0) | 30(36.0) | χ²=4.5, df=2  p₁= 0.10 |
|  | 321(50.3) | 49(55.1) |  |
|  | 30(4.7) | 8(9.0) |  |
| Exposure to SHS within the last 7 days n (%) |  |  |  |
| Yes | 115(18.2) | 18(20.7) | χ²=0..32, df=2  p₁= 0.57 |
| No | 517(81.8) | 69(79.3) |  |
| Exposure to SHS within the last 30 days (n%) |  |  |  |
| Yes | 149(23.6) | 23(25.8) | χ²=0.21, df=2  p₁= 0.64 |
| No | 482(76.4) | 66(74.2) |  |
| Median knowledge on health risk of exposure to SHS | 9 | 10 | p₁=0.09 |
| Median attitude on exposure to SHS | 11 | 11 | p₂=0.12 |
| Median attitude on right to smoke free living | 5 | 5 | p₂=0.79 |
| Median attitude on women empowerment | 5 | 5 | p₂=0.24 |
| Median observed evidence on smoking | 0 | 0 | p²=0.86 |

*Data missing: education 1, exposure to SHS within 7 days 8, exposure to SHS within 30 days 7*

*χ² = Chi-square, df=degree of freedom,* p₁ =*Chi-square test, between completers and non-completers*

P₂*= Mann-Witney* U *Test, between completers and non-completers.*
