# Supplementary material for: The impact of multiple interventions to reduce household exposure to second-hand tobacco smoke among women: a cluster randomized controlled trial in Kalutara district, Sri Lanka
Source: BMC Public Health. 2017 Oct 16;17:810. doi: 10.1186/s12889-017-4820-8 (PMC5644088; doi:10.1186/s12889-017-4820-8)
Supplement: Supplementary file 2 — Analysis of difference for primary and secondary outcomes between intervention and control clusters. (DOCX 14 kb) [file 12889_2017_4820_MOESM2_ESM.docx]

**Additional file 2**

**Table S2: Analysis of difference for primary and secondary outcomes between intervention and control clusters**

| **Outcomes** | **Mean difference**  **Baseline**  **Follow-up** | **95% CI**  **Baseline**  **Follow-up** | **significance** |
| --- | --- | --- | --- |
| Exposure to SHS in their households within last 7 days | 0.01  -0.06 | -0.07-0.09  -0.13-0.01 | 0.73  0.05 |
| Exposure to SHS in their households within last 30 days | 0.03  -0.12 | -0.06-0.11  -0.40-0.78 | 0.65  0.04 |
| knowledge on health risk of exposure to SHS | 0.06  1.06 | -0.39-0.52  0.74-1.37 | 0.77  0.004 |
| Attitude on exposure to SHS | 0.29  0.56 | -0.35-0.92  -0.10-1.22 | 0.36  0.04 |
| Attitudes on right to smoke free living | 0.21  0.47 | -0.21-0.62  0.06-0.87 | 0.31  0.02 |
| Attitudes on women empowerment against SHS | 0.01  1.34 | -0.57-0.60  0.79-1.89 | 0.96  0.008 |
